# PHA665752, a small-molecule inhibitor of c-Met, inhibits hepatocyte growth factor-stimulated migration and proliferation of c-Met-positive neuroblastoma cells

**DOI:** 10.1186/1471-2407-9-411

**Published:** 2009-11-25

**Authors:** Hal E Crosswell, Anindya Dasgupta, Carlos S Alvarado, Tanya Watt, James G Christensen, Pradip De, Donald L Durden, Harry W Findley

**Affiliations:** 1 Division of Pediatric Hematology/Oncology, Children's Hospital and University Medical Group of the Greenville Hospital System, Greenville, SC 29605, USA; 2 Division of Pediatric Hematology/Oncology and Bone Marrow Transplantation, AFLAC Cancer Center and Blood Disorders Service, Department of Pediatrics, Emory University School of Medicine, Atlanta, GA 30022, USA; 3 Cancer Biology, Pfizer Inc., La Jolla, CA 92121, USA

## Abstract

**Background:**

c-Met is a tyrosine kinase receptor for hepatocyte growth factor/scatter factor (HGF/SF), and both c-Met and its ligand are expressed in a variety of tissues. C-Met/HGF/SF signaling is essential for normal embryogenesis, organogenesis, and tissue regeneration. Abnormal c-Met/HGF/SF signaling has been demonstrated in different tumors and linked to aggressive and metastatic tumor phenotypes. *In vitro *and *in vivo *studies have demonstrated inhibition of c-Met/HGF/SF signaling by the small-molecule inhibitor PHA665752. This study investigated c-Met and HGF expression in two neuroblastoma (NBL) cell lines and tumor tissue from patients with NBL, as well as the effects of PHA665752 on growth and motility of NBL cell lines. The effect of the tumor suppressor protein PTEN on migration and proliferation of tumor cells treated with PHA665752 was also evaluated.

**Methods:**

Expression of c-Met and HGF in NBL cell lines SH-EP and SH-SY5Y and primary tumor tissue was assessed by immunohistochemistry and quantitative RT-PCR. The effect of PHA665752 on c-Met/HGF signaling involved in NBL cell proliferation and migration was evaluated in c-Met-positive cells and c-Met-transfected cells. The transwell chemotaxis assay and the MTT assay were used to measure migration and proliferation/cell-survival of tumor cells, respectively. The PPAR-γ agonist rosiglitazone was used to assess the effect of PTEN on PHA665752-induced inhibition of NBL cell proliferation/cell-survival and migration

**Results:**

High c-Met expression was detected in SH-EP cells and primary tumors from patients with advanced-stage disease. C-Met/HGF signaling induced both migration and proliferation of SH-EP cells. Migration and proliferation/cell-survival were inhibited by PHA665752 in a dose-dependent manner. We also found that induced overexpression of PTEN following treatment with rosiglitazone significantly enhanced the inhibitory effect of PHA665752 on NBL-cell migration and proliferation.

**Conclusion:**

c-Met is highly expressed in most tumors from patients with advanced-stage, metastatic NBL. Furthermore, using the NBL cell line SH-EP as a model, PHA665752 was shown to inhibit cMet/HGF/SF signaling *in vitro*, suggesting c-Met inhibitors may have efficacy for blocking local progression and/or metastatic spread of c-Met-positive NBL *in vivo*. These are novel findings for this disease and suggest that further studies of agents targeting the c-Met/HGF axis in NBL are warranted

## Background

Children with metastatic neuroblastoma (NBL) who are older than 12 months at diagnosis typically have a poor outcome despite modern multimodal therapy. In most of these patients, the tumor has unfavorable biological characteristics such as MYCN oncogene amplification, deletions of the short arm of chromosome 1, deletions of 11q, expression of the TrkB neurotrophin receptor and its ligand, and/or other cytogenetic and molecular abnormalities [1]. However, recurrent disease and poor outcome may also occur in children with tumors lacking these adverse biological features. This suggests that other as yet undefined factors contribute to an aggressive neuroblastoma phenotype.

C-Met is a tyrosine-kinase receptor for hepatocyte growth factor/scatter factor (HGF/SF), and both receptor and ligand are expressed in a number of different tissues [2,3]. Binding of activated HGF/SF to the extracellular domain of c-Met causes multimerization of the receptor and phosphorylation of tyrosine residues at the juxtamembrane and cytoplasmic regions. This is followed by recruitment and phosphorylation of multiple adaptor proteins, i.e. Grb2, Gab1, SHC, and c-Cbl, as well as activation of signaling molecules such as phosphatidylinositol-3-OH kinase (PI3-K), PLC-γ, STAT3, phospholipase C-γ, Erk 1 and 2, and FAK [4-8]. PI3-K and Erk are necessary not only for c-Met-mediated regulation of cell motility, adhesion, and invasion, but also for control of cell survival (via the Akt pathway) and mitogenesis [9].

C-Met/HGF/SF signaling is essential for normal cell proliferation, migration, angiogenesis, embryogenesis, organogenesis, and tissue regeneration. Additionally, there is now considerable evidence suggesting that aberrant c-Met/HGF/SF signaling, resulting from mutation or overexpression of the c-Met proto-oncogene and/or its ligand, plays a major role in tumorigenesis, invasion, and metastatic spread in many human tumors [10,11]. Tumor lines with mutated c-Met or overexpressed c-Met and/or HGF/SF [12-14] are tumorigenic *in vitro *and *in vivo*; and tumor cells transfected with c-Met and HGF/SF are capable of forming tumors with an invasive and metastatic phenotype in the nude mice [15]. HGF/SF transgenic mice develop a wide array of mesenchymal- and epithelial-derived tumors which overexpress HGF/SF and c-Met [16]. Similarly, transgenic mice carrying the TPR-MET gene (coding for an oncogenic TPR-MET fusion protein) develop Met-driven T-cell lymphomas [17]. Expression of c-Met and/or HGF has been detected in cell lines established from pediatric tumors including rhabdomyosarcoma, osteogenic sarcoma, and neuroblastoma [12,18,19]. Furthermore, abnormal c-Met/HGF/SF signaling has been noted in different types of malignant solid tumors and correlates with advanced stages and poor prognosis [20,21]. More recently, overepression of c-Met and HGF has also been observed in hematopoietic malignancies, i.e. multiple myeloma and adult T- cell leukemia [22,23].

Given the oncogenic role of aberrant c-Met/HGF/SF signaling, c-Met has become an attractive therapeutic target [2,24]. One way to effectively block c-Met signaling is by inhibiting its catalytic activity with small-molecule inhibitors. One such inhibitor is PHA665752, a highly selective c-Met inhibitor which competitively inhibits binding of ATP to the tyrosine kinase domain of c-Met. *In vitro*, PHA665752 inhibits constitutive and HGF/SF-stimulated c-Met phosphorylation, cell growth, motility, and migration of different tumor cell lines [22,25-27]. At nanomolar concentrations, it induces massive apoptosis of gastric carcinoma cells with amplified c-Met [28]. *In vivo*, daily administration of PHA665752 into athymic mice blocked c-Met phosphorylation and caused growth inhibition of tumor xenografts [14,27].

Phosphatase and tensin homologue (PTEN) is a tumor suppressor protein that modulates several cell functions including proliferation, survival, migration, and tumor-induced angiogenesis mainly by antagonizing PI3K-Akt signaling [29-31]. Mutation or loss of PTEN function has been observed in some cases of NBL and other solid tumors and results in a more aggressive tumor phenotype [32]. In contrast, upregulation of PTEN inhibits proliferation of malignant solid tumor cells *in vitro *[33,34].

Studies of the expression and role of c-Met expression in NBL have thus far been limited to cell lines (12, 18,19). We here report for the first time that c-Met is expressed at high levels in advanced-stage, primary NBL tumor tissue. Furthermore, we describe the effects of a small-molecule c-Met inhibitor, PHA665752, on HGF-induced migration and proliferation of NBL cells. We also report the effect of augmented PTEN expression on PHA665752-mediated inhibition of c-Met-HGF/SF signaling in this tumor.

## Methods

### Cell lines and tumor tissue

The human NBL lines SH-EP and SH-SY5Y were used to determine HGF and c-Met gene expression and to assess the effects of PHA665752 (Pfizer) on Met/HGF-induced proliferation and migration of tumor cells. Both cell lines have a single copy of the MYCN oncogene [35]. These cells lines were also chosen because SH-EP expresses c-Met, whereas SH-SY5Y is c-Met negative [19]. Additionally, SH-EP cells show significant proliferative and migratory responses to HGF. SKN-AS served as positive control for HGF expression in qRT-PCR and immunoblot assays. Cells were grown in Dulbecco's modified Eagle medium supplemented with 10% fetal bovine serum (FBS) and 1% penicillin/streptomycin (Sigma Chemical Co., St. Louis, Mo.). Primary tumor samples were obtained during diagnostic surgery from patients treated at Children's Healthcare of Atlanta hospitals following parental informed consent and Emory University IRB approval. RNA was extracted for quantitative RT-PCR studies of c-Met expression as described below.

### Transfection experiments

SH-SY5Y cells were plated in 6-well plates and transiently transfected with increasing concentrations of full-length human c-Met cDNA in a pMOG vector or vector alone (a kind gift from G. Vande Woude, Van Andel Research Institute, Grand Rapids, MI) using Lipofectamine Plus (Invitrogen, Carlsbad, CA). At 48 hours, cells were washed once with PBS and harvested.

### Antibodies and reagents

C-Met inhibitor PHA665752 (Pfizer, Inc., La Jolla, CA) was dissolved in DMSO. LY294002 (in DMSO; Calbiochem) and PD98059 (aqueous solution; Calbiochem) were used at the concentrations described below. Antibodies to c-terminus of c-Met (C-12) and Erk (C-16) were from Santa Cruz Biotechnology (Santa Cruz, CA); p-Erk 1 and 2 (p44/42), p-Akt (Ser^473^), Akt and phosphospecific c-Met (Tyr ^1234/1235^) were from Cell Signaling Technology (Beverly, MA); mouse polyclonal HGF antibody was from R&D Systems (Minneapolis, MN). Rosiglitazone, a PPAR-γ agonist and inducer of PTEN, was obtained from Cayman Chemical (Ann Arbor, MI) [34]. Recombinant human HGF was from PeproTech (Rocky Hill, NJ).

### Reverse transcription and quantitative real-time polymerase chain reaction (qRT-PCR)

qRT-PCR was used to assay for c-Met and HGF mRNA expression in cell lines, conditioned media, and tumor tissue. Briefly, total RNA from neuroblastoma lines was extracted using the RNAeasy kit (Qiagen, Valencia, CA). The corresponding cDNAs were synthesized by Quantitech Reverse Transcription kit (Qiagen). Quantitative RT-PCR assays for c-Met and HGF were set up with gene specific primers using Quantitect SYBR-Green PCR kit (Qiagen) and executed on a 7500 Real-Time PCR instrument (Applied Biosystems, Foster City, CA). Mean expression values for each mRNA sample were normalized against its GAPDH mRNA level. The c-MET and GAPDH primers were purchased as proprietary SYBR-Green validated primer sets (Qiagen).

The HGF primers were designed by us and synthesized by the Microchemical Facility, Emory University, using the following sequences:

HGF (forward): 5'CTAGATCTTTCCAGTTAATCACACAAC 3'

HGF (reverse): 5'TTCGGAGTCAGTGCCTAAAAGAG 3'

PCR cycling conditions consisted of an initial enzyme activation step at 95° for 15 min followed by a total of 40 cycles including denaturation at 94°C for 15 sec, annealing at 55°C for 30 sec. and final extension at 72°C for 34 sec. The extension step served for fluorescence detection. Detection of gene specific amplicons was verified by dissociation curve analyses. The SH-SY5Y line was designated as the calibrator to quantitate both HGF and c-MET mRNA expression levels.

### Proliferation/cell-survival assay

Cells were plated at 1-10^4 ^cells/well in 96-well plates and grown in presence of factors described below. After 72 hours of growth, cells were washed once with PBS and analyzed by the MTT proliferation/viability assay (Invitrogen, Carlsbad, CA). Effects on cell viability were further confirmed by trypan-blue assay. Student's t-test was used to determine significant differences among means for independent proliferation/cell-survival assays performed in triplicate, as well as to evaluate the significance of differences in c-Met expression between stage 3-4 *vs *stage 1-2 primary tumors.

### Migration assay

Cell-migration was assessed using a trans-well chemotaxis assay as previously described [36,37]. In brief, bottom membranes of transwell chambers of diameter 6.5 mm, 8 um pore size (Costar Corp., Cambridge, MA) were coated with vitronectin (Sigma Chemical Co., St. Louis, Mo) at 10 ug/ml for 1 hour and inserted in 12-well plates. Cells were treated with specific inhibitor (PHA66572 or other inhibitor) for 60 minutes and then washed. Treated cells were plated in equal numbers at 1-2 × 10^5 ^in the upper chamber and allowed to migrate across the membrane toward 200 ng/ml HGF (bottom chamber) for 6 hours at 37°C, 5% humidified CO_2_. Migration was quantified either with 1% crystal violet and manual counting or by automated counting of nuclear-stained cells. Student's t-test was used to determine significant differences among means for independent migration experiments performed in triplicate.

### Immunoblot analysis

Whole cell lysates were prepared by washing cells once with ice-cold PBS and adding 400 uL of lysis buffer. Protein was quantitated (Bio-Rad Laboratories, Hercules, CA), and equal amounts of protein were resolved by SDS-PAGE and transferred to nitrocellulose. Membranes were blocked with 5% non-fat milk and probed overnight with antisera-specific antibodies at 1:500 dilution. Incubation buffer consisted of either TBST (10 mM Tris [pH 7.6], 50 mM NaCl, and 0.1% Triton X-100) containing 5% non-fat milk or 5% bovine serum albumin (for phosphorylated antibodies). Bound primary antibodies were visualized after washing and probing with appropriate horseradish peroxidase-conjugated detection antibodies at 1:2000 dilution for one hour, using signal-enhanced chemiluminescence (SuperSignal Chemiluminescent substrate, Pierce, Rockford IL). Membranes were stripped and reprobed after washing and blocking. Equal loading was determined by probing for β-actin (Sigma Chemical Co., St. Louis, Mo).

## Results

### c-Met and HGF expression in NBL cell lines

**Figure 1:**
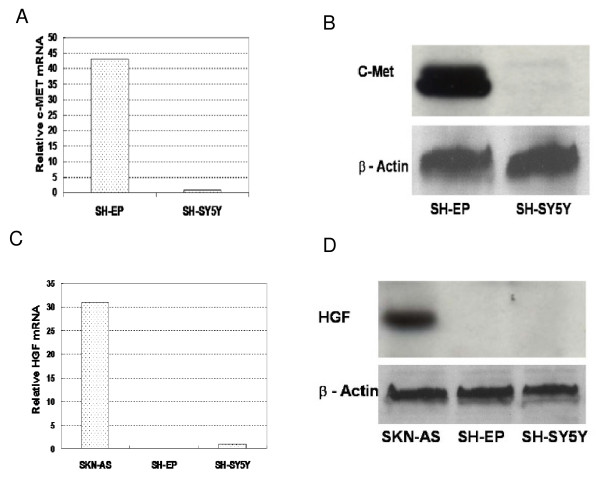
**Expression of c-Met and HGF by SH-EP and SH-SY5Y neuroblastoma cell lines**. Quantitative RT-PCR assay for c-Met (A) and HGF (C) was performed in triplicate using relative quantification, with expression values normalized against GAPDH. Western blots for the 140 kD c-Met beta-chain (B) and 82 kD HGF (D) proteins, with SKN-AS neuroblastoma cell line as the positive control for HGF protein expression. β-Actin serves as loading control.

SH-EP cells expressed significantly more c-Met than did SH-SY5Y cells at both the mRNA and protein level (Figure 1A/B). In contrast, both SH-SY5Y and SH-EP cells were either very low or negative for HGF mRNA, respectively. Both lines lacked detectable HGF protein (Figure 1C/D). Conditioned media from unstimulated and vitronectin-stimulated SH-EP cells did not contain measurable amounts of HGF (data not shown).

### PHA665752 inhibits HGF-stimulated migration and proliferation/cell-survival of c-Met-positive neuroblastoma cells

**Figure 2:**
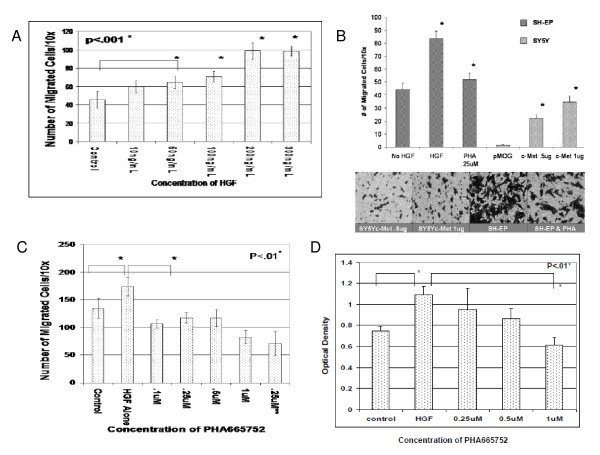
**PHA665752 inhibits SH-EP cell migration and proliferation/cell-survival**. (A) Migration of SH-EP cells on vitronectin (10 ug/ml) was assessed in the transwell chemotaxis assay with increasing concentration gradients of HGF. (B) Migration of SH-EP vs. SY5Y was assessed on vitronectin (10 ug/ml) in the transwell chemotaxis assay in the presence of 200 ng/ml HGF either with or without pre-incubation with PHA665752. SY5Y cells were transiently transfected with empty pMOG vector or with either 0.5 ug or 1 ug of c-Met cDNA; inserts show photomicrographs (20× magnification) of migrated cells under specified conditions. (C) HGF-induced migration of SH-EP cells was measured in the transwell chemotaxis assay after either 60 minute pre-incubation or during continuous exposure(**) to PHA665752. (D) HGF-induced proliferation/cell-survival of SH-EP cells was measured by MTT assay either with or without increasing concentrations of PHA665752 over 72 hrs. Data represent mean +/- SD for triplicate independent experiments.

In a semi-quantitative wound-healing assay, we found that SH-EP cells migrated in response to HGF, and this response was greater than that observed with low-c-Met expressing SH-SY5Y cells (data not shown). In the transwell chemotaxis assay, SH-EP cells demonstrated a dose-dependent migration response to HGF (Figure 2A). Transfection experiments showed that only SH-SY5Y cells transfected with c-Met migrated in response to HGF; furthermore, response correlated with the amount of transfected c-Met DNA (Figure 2B). In the MTT-proliferation assay, SH-EP cells showed a proliferative response to HGF in both 72-hr (Figure 2D) and 7-day growth assays (data not shown). PHA665752 inhibited both HGF-mediated migration (Figure 2C) and proliferation/cell-survival (Figure 2D) in a dose-dependent manner. IC50 values for PHA665752-induced inhibition of migration and proliferation ranged from 0.25-0.5 uM.

### PHA665752 inhibits c-MET/HGF migration and signaling via the MAPK pathway

**Figure 3:**
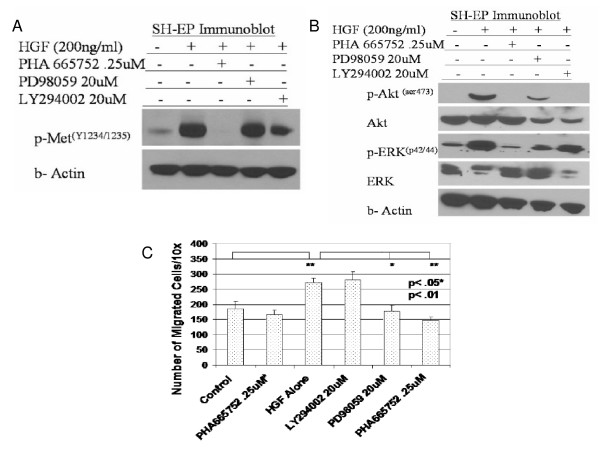
**PHA665752 inhibits HGF-induced migration through blockade of MAPK pathway**. (A) SH-EP cells were pretreated for 60 minutes with PHA665752, PD98059 or LY294002 as shown, washed, and analyzed by transwell chemotaxis assay in presence of HGF (200 ng/mL). (B, C) SH-EP cells were pretreated with inhibitors for 60 minutes, followed by addition of HGF (200 ng/mL for 15 mins) and immunoblotting of cell lysates. (in Figure 3C, # indicates incubation with vitronectin alone without HGF; **+ **indicates addition of HGF). Data represent mean +/- SD for triplicate independent experiments.

PHA665752 completely abrogated HGF-mediated activation of c-Met in SH-EP cells as determined by p-Met^(Y1234/1235) ^levels (Figure 3A); PHA665752 also completely blocked HGF-induced phosphorylation of both MAPK and PI3-K downstream signaling, using p-Erk 1/2 and p-Akt^(ser473) ^levels as surrogate markers, respectively (Figure 3B). Experiments to further characterize the migration-signaling pathways inhibited by PHA665752 showed that PD98059, a MAPK inhibitor, suppressed HGF-activated migration to a similar extent as PHA665752 alone, whereas LY294002, a PI3-K inhibitor, had no effect on migration (Figure 3C).

### PTEN induction augments the inhibitory effect of PHA66572 on HGF-mediated proliferation and migration

**Figure 4:**
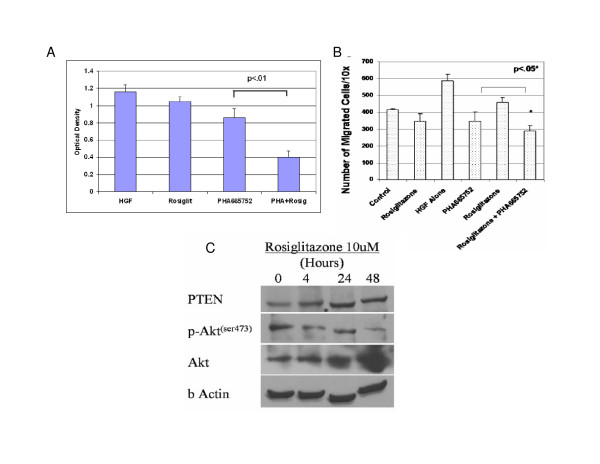
**PHA665752-mediated inhibition of proliferation and migration is augmented by PTEN-agonist rosiglitazone**. (A) SH-EP cells were cultured with HGF (200 ng/ml) and either PHA665752 (0.25 uM), PPAR-γ agonist rosiglitazone (10 uM), or both PHA665752 and rosiglitazone for 72 hrs; HGF alone served as a control. Proliferation/cell-survival was analyzed by MTT assay. (B) SH-EP cells were exposed to 10 uM rosiglitazone and 0.25 uM PHA665752 overnight, washed, and assessed for migration in the presence of 200 ng/ml HGF in the transwell chemotaxis assay (**+ **indicates addition of HGF). (C) PTEN and p-Akt expression were measured in lysates of SH-EP cells after exposure to 10 uM rosiglitazone for stated time period. Data represent mean +/- SD for triplicate independent experiments.

To evaluate PTEN's ability to potentiate the effects of PHA665752 on c-Met/HGF signaling, we treated SH-EP cells with rosiglitazone, an inducer of PTEN expression [34]. To assess rosiglitazone's effect on proliferation, SH-EP cells were grown in the presence or absence of HGF, with or without rosiglitazone. Rosiglitazone had no effect on SH-EP proliferation in the absence of HGF, although it somewhat reduced HGF-stimulated proliferation (Figure 4A). Importantly, combined PHA665752 and rosiglitazone was significantly (p < .01) more inhibitory for HGF-stimulated SH-EP cell proliferation than was either agent alone HGF (Figure 4A). Furthermore, migration of PHA665752-treated SH-EP cells was significantly reduced when pretreated with rosiglitazone, demonstrating that rosiglitazone augments the migration-inhibitory effects of PHA665752, although the magnitude of this effect was less than that on NBL cell growth (Figure 4B). Rosiglitazone's inhibitory effects on HGF-stimulated proliferation/cell-survival and migration correlated with greater than two-fold inductionof PTEN protein as shown by immunoblotting (Figure 4C).

### Expression levels of c-Met mRNA in primary NBL tumor tissue correlates with advanced clinical stage

**Figure 5:**
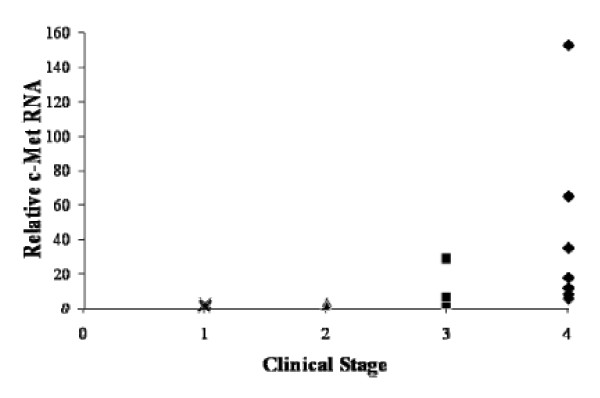
**Advanced clinical stage in patients with neuroblastoma correlates with high c-Met mRNA levels**. mRNA was isolated from banked tumor samples from patients with localized or metastatic neuroblastoma. Quantitative RT-PCR for c-Met was performed using GADPH as internal control and the SH-EP line as a positive control. Values are expressed relative to the SH-SY5Y line, which is set equal to one; thus, SH-EP c-Met value = 15.8. c-Met values represent the mean of triplicate samples and are plotted according to INSS clinical stage. Median c-Met values for each stage are 0.87 (stage 1), 2.6 (stage 2), 5.2 (stage 3), and 17.5 (stage 4). c-Met values for stage 3-4 tumors are significantly higher (p = 0.048) than values for stage 1-2 tumors.

To evaluate the possible clinical significance of c-Met expression in NBL, we used quantitative RT-PCR to determine c-Met expression levels in mRNA collected from 20 primary neuroblastoma tumors at different clinical stages. Seven tumors were stage 4, five were stage 3, two were stage 2, and six were stage 1. Tumors from patients with more advanced clinical stages (stages 3 and 4) generally had higher c-Met expression levels than did tumors from patients with stages 1 and 2. Four of six stage-4 tumors and one of five stage-3 tumors had c-Met values greater than the corresponding value for SH-EP cells, whereas no stage-1 or 2 tumors had c-Met values in this range (Figure 5).

## Discussion

In this study, we investigated the expression and role of c-Met in NBL. We found that primary tumor cells from patients with clinically aggressive, advanced-stage NBL expressed high-levels of c-Met. Tumor cells from patients with metastatic tumor (stage 4) or locally advanced tumor (stage 3) expressed significantly higher c-Met levels than those from patients with localized disease, i.e. stages 1 and 2. This novel finding strongly suggests that inhibiting c-Met may have therapeutic value in this disease. Accordingly, we chose to test a c-Met inhibitor (PHA665752) with high specificity and potency for blocking c-Met function.

To evaluatethe efficacy of PHA66572 on the migration and proliferation of c-Met expressing NBL cells, we used a c-Met-positive NBL line (SH-EP) as an *in vitro *model. We found that activation of the c-Met/HGF/SF pathway resulted in increased migration and proliferation-survivalof SH-EP cells but not of c-Met-negative SH-SY5Y cells. This is the first report of the effects of specifically blocking c-Met in NBL cells. Our results agree with those reported by Hecht et al, who showed that exposure of c-Met-expressing NBL cell lines to exogenous HGF resulted in c-Met phosphorylation and induction of migration [19]. These investigators were also able to inhibit migration of NBL cells in Matrigel using an HGF-specific neutralizing antibody and MAPK/ERK inhibitors such as PD98059, respectively. Similarly, Hov et al reported that c-Met activation stimulated both proliferation and migration of myeloma cells *in vitro *[22]. A role for c-Met in NBL cell migration is further suggested by our finding that HGF triggered migration of SH-SY5Y cells only after transfection with c-Met.

In our experiments to evaluate the effects of PHA665752, we found that this small-molecule inhibitor was able to block HGF-induced phosphorylation of both c-Met and downstream signaling proteins Akt and Erk 1/2 (p44/42). Studies of myeloma and carcinoma cells have yielded similar results [22,26,27]. However, in the latter studies, tumor cells were exposed to PHA665752 continuously, whereas in the present study NBL cells were only briefly exposed to low concentrations of this agent. This suggests that NBL cells may be more sensitive to c-Met targeting than some other c-Met expressing tumors.

We also found that PHA665752 showed a marked dose-dependent inhibitory effect on the HGF/c-Met pathway of proliferation and migration in c-Met-expressing NBL cells. These inhibitory effects appeared to be specific for HGF-stimulated proliferation/migration, since PHA665752 had no significant effects on these parameters in the absence of HGF stimulation. Thus, PHA665752 could potentially inhibit HGF-stimulated tumor proliferation/migration resulting from either paracrine (i.e. tumor microenvironment) or autocrine exposure to HGF. Furthermore, brief exposure to PHA665752 completely blocked downstream signaling *via *the PI3-K/Akt and MAPK/Erk pathways, agreeing with results from studies in several types of carcinoma [25,27]. Additional studies with MAPK/Erk and PI3-K/Akt pathway-specific inhibitors suggested that PHA665752 can inhibit c-Met/HGF/SF-stimulated migration and proliferation/survival *via *the MAPK/Erk and PI3-K/Akt pathways, respectively.

Since PHA665752 inhibits the PI3-K/Akt pathway, we hypothesized that its effect might be enhanced by rosiglitazone, an inducer of PTEN expression. Indeed, combined PHA665752 and rosiglitazone induced significantly greater inhibition of both HGF-stimulated proliferation/cell-survival and migration in c-Met-expressing NBL cells than did PHA665752 alone. This finding suggests that the combination of PTEN-inducing agents with small-molecule inhibitors or drugs that block c-Met/HGF/SF signaling may have an augmented anti-tumor effect.

Although we do not expect PHA665752 will be suitable for clinical use due to its tendency to form pulmonary precipitates in animal studies [17], we believe this agent provides an excellent tool for studying c-Met function in NBL due to its high specificity and activity. Finally, our study supports the notion that c-Met blockade, either through derivatives of PHA665752 with higher bioavailability or through other agents targeting this receptor, deserves further study as a potential therapeutic strategy for NBL.

## Conclusion

Elevated c-Met expression is more commonly observed in primary NBL tumor tissue from patients with metastatic tumors. Furthermore, the small-molecule inhibitor PHA665752 is capable of antagonizing HGF-induced migration and proliferation/survival of c-Met-expressing NBL cells, an effect which is enhanced by upregulation of PTEN. These are novel findings in NBL and suggest a therapeutic potential for targeting c-Met in this tumor.

## Competing interests

One of the co-authors (JGC) is employed by Pfizer, which provided the drug under study (PHA665752).

## Authors' contributions

HEC, HWF, and CSA designed the study and wrote the manuscript. HEC also performed the migration, proliferation and transfection experiments with the assistance of TW, DLD and PD. AD grew the cell lines and performed qRT-PCR studies on tumor tissue, as well as assisted in MTT/viability assays for proliferation/cell-survival. JGC kindly provided PHA665752 and advice regarding experimental design. All authors read and approved the manuscript.

## Pre-publication history

The pre-publication history for this paper can be accessed here:

http://www.biomedcentral.com/1471-2407/9/411/prepub
